# Assessment of self-reported adherence among patients with type 2 diabetes in Matlala District Hospital, Limpopo Province

**DOI:** 10.4102/phcfm.v8i1.900

**Published:** 2016-07-28

**Authors:** Sadeen A. Adegbola, Gert J.O. Marincowitz, Indiran Govender, Gboyega A.O. Ogunbanjo

**Affiliations:** 1 Department of Family Medicine & Primary Health Care, University of Limpopo, South Africa; 2 Department of Family Medicine, Sefako Makgatho Health Sciences University, South Africa

## Abstract

**Introduction:**

Complications associated with Diabetes Mellitus are a burden to health services, especially in resource poor settings. These complications are associated with substandard care and poor adherence to treatment plans. The aim of the study was to assess the self-reported adherence to treatment amongst patients with type 2 diabetes in Matlala District Hospital, Limpopo Province.

**Methods:**

This cross-sectional study used convenience sampling with a standardised, validated questionnaire. Data were collected over 4 months, and Microsoft Excel was used for data capturing.

**Results:**

We found that 137 (70%) of the participants considered themselves adherent to their diabetes medication. Younger age (*p* = 0.028), current employment (*p* = 0.018) and keeping appointment were factors significantly associated with adherence. Reasons given for poor adherence were that the clinic did not have their pills (29%), they had forgotten to take their medication (16%) and gone travelling without taking enough pills (14%). Reasons given for poor adherences to a healthy lifestyle were being too old (29%), 22% had no specific reason, 13% struggled to motivate themselves and 10% simply forgot what to do. Sixty-eight percent of the adhered participants recommended the use of medication at meal times, 14% set a reminder, and 8% used the assistance of a treatment supporter

**Conclusions and recommendations:**

The study revealed a higher than expected reported level of adherence to diabetes treatment. Further research is needed to assess whether self-reported adherence corresponds to the metabolic control of the patients and to improve services.

## Introduction

Diabetes mellitus is a silently progressive, but serious, health condition. Globally, 386.7 million people suffer from diabetes with a prevalence of 8.3%. This is expected rise to 592 million people by 2035.^1^ A large portion of this increase will occur in developing countries, arising from growth and an increased life expectancy of the population, as well as the lifestyle changes associated with increasing trends towards urbanisation, including unhealthy diets, obesity and a sedentary lifestyle, resulting in late-onset diabetes. The International Diabetes Federation estimated that in 2014, 4.9 million people died of diabetes-related deaths worldwide,^1^ and furthermore, diabetes is the sixth leading cause of death in South Africa.^2^ Based on available epidemiological data, approximately 1–1.5 million South Africans are considered to suffer from diabetes.^3^

Patient adherence to diabetes medication plays an integral role in reducing the healthcare costs resulting from poor adherence, including repeated hospitalisations, the management of complications as well as the subsequent rehabilitation process. Identifying which patients are at the greatest risk of non-adherence is an important first step towards developing interventions that could improve adherence.^4,5,6^ Diabetes is considered one of the most emotionally demanding chronic diseases, with its management requiring self-monitoring of blood glucose, dietary modifications, exercise and adherence to treatment regimens.^7^ Many of the complications associated with diabetes can be delayed or prevented by better management and self-care, with improved glycaemic control through diet, exercise and/or taking insulin or oral diabetes medications.^8,9^

This study was conducted at the Matlala District Hospital, a 288-bed hospital with outreach to seven primary health clinics, located in Sekhukhune district, Limpopo. It serves a population of 74 867 people and an average of 120 patients attend the outpatient department daily. According to the outpatient register, more than a third of these patients are on medication for chronic conditions (excluding patients on antipsychotics) of which hypertension is the highest, followed by diabetes mellitus (7.6%). From April 2008 to March 2009, monthly averages of 139 patients with diabetes mellitus were seen at the hospital outpatient department, and of this number an average of eight were newly diagnosed patients with diabetes. Whilst working in the medical wards, the main researcher (S.A.A.) had the impression that many of the subsequent outpatient admissions were because of complications of diabetes, a likely consequence of poor or non-adherence to treatment.

This prompted the researchers to investigate the level of adherence to treatment as reported by patients with type 2 diabetes seen at Matlala District Hospital and to identify the reasons for poor or non-adherence to treatment and lifestyle changes.

## Methods

A cross-sectional study was carried out where all patients with type 2 diabetes attending the outpatient department of Matlala District Hospital, who gave written informed consent, were included in the study. Patients with type 1 diabetes and patients who had been diagnosed less than a month previously were excluded.

A prospective sample size of *N* = 196 was calculated using the formula:

$$n=Nz^{2}pq/e^{2}\{N-1\}+z^{2}pq$$ [Eqn 1]

to ensure a statistical power of > 95% [*n* = sample size; *N* = population size (500); *Z* = critical value (1.96); *p* = estimated proportion {30% = 0.3}; *q* = 1 – *p* and *e* = level of precision (±5% = 0.05)]. The number of patients with type 2 diabetes at Matlala Hospital was estimated to be 500, and the adherence was estimated to be 30%. The low estimate was based on the low socio-economic environment and literature review that gave levels of 50%.

Data were collected from 1 December 2009 to 30 March 2010 by a trained interviewer who administered a structured questionnaire in the local vernacular. The questionnaire, adapted from studies on adherence in hypertensive patients^10^ as well as tuberculosis patients’ reasons for defaulting treatment^11^, was limited to 22 questions and took an average of 10 minutes to complete. Adherence to medication and lifestyle changes was assessed by asking participants to recall the taking of medication and lifestyle changes (exercise) for the week preceding the visit to the hospital. The responses were categorised as ‘always’, ‘frequently’, ‘only when I experienced diabetic symptoms’ and ‘never’. The translation of the questionnaire was verified by a language expert, and a pilot study was conducted before data collection to test the reliability and validity of the questionnaire. Data were captured on an Excel spread sheet and interpreted with cross-tabulation to determine association.

### Ethical considerations

The study was approved by the MEDUNSA Research and Ethics Committee, University of Limpopo. Participants gave written consent and were assured of confidentiality.

## Results

### Demographic Characteristics

Of the 196 participants, 137 (70%) adhered to diabetes treatment and 59 (30%) reported non-adherence to treatment (Table 1). Two-thirds of them, 127 (65%), reported that they heeded lifestyle advice and 69 (35%) admitted non-adherence.

**TABLE 1:** Demographic characteristics.

Participants	Number
**Sex**	
Women	143
Men	53
**Age**	
> 60	100
50–59	63
< 50	33
**Marital status**		
Married	130
Widowed	39
Separated/divorced	13
Single	14
**Family size**	
1–3 household members	40
4–6 household members	94
7–9 household members	35
> 10 household members	17
**Education**	
No formal education	90
Primary education	61
Secondary education	34
Tertiary education	11
**Employment**	
Unemployed	170
Employed	12
Self-employed	12
Informal sector	2

*Source*: Author’s own work

The majority of the unemployed participants (140) indicated a grant as a source of income, while 17 said that they received financial support from their families and 13 mentioned other sources of income. With regards to household income, 134 participants (68%) had a household income of R1000−R1999 per month, and 38 (19%) survived on an income of less than R1000 per month.

### Adherence

Age below 50 years (*p* = 0.028), being employed (*p* = 0.018) and the keeping of appointments (*p* = 0.001) were significantly associated with being adherent to treatment, while gender (*p* = 0.441), marital status (*p* = 0.294), the level of education (*p* = 0.567), the distance travelled (*p* = 0.452) or the drug regimen (*p* = 0.928) were not significantly associated with adherence (Table 2).

**TABLE 2:** Adherence and the demographic characteristics of the participants.

Characteristics	Adherence		*p*-value

	Yes (%)	No (%)	
**Age**			
< 50	28 (20)	5 (8)	0.028
≥ 50	109 (80)	54 (92)	
**Level of education**			
None/primary	104 (76)	47 (79)	0.567
Secondary/tertiary	33 (24)	12 (21)	
**Employment status**			
Employed	23 (17)	3 (5)	0.018
Unemployed	114 (83)	56 (95)	
**Distance travel to facility, km**			
≤ 10 km	90 (66)	42 (71)	0.452
≥ 10 km	47 (34)	17 (29)	
**Keeping of appointments**			
Yes	135 (98)	52 (88)	0.001
No	2 (2)	7 (12)	

*Source*: Author’s own work

Table 3 presents reasons for poor adherence to diabetic medication. The most important reasons that emerged were the following: (1)the clinic did not have participants’ pills (29%), (2) participants forgetting to take their medication (16%) and (3) participants who forgot to take enough medication with them while travelling (14%).

**TABLE 3:** Reasons for poor adherence to diabetic medication.

Reasons	Number of respondents	% of respondents
The clinic did not have my pills	17	29
I forgot	9	16
I travelled to visit and did not have enough pills	8	14
My medication was finished	6	10
I do not have food to eat before I take my pills	5	9
I am taking care of a sick family member	4	7
I do not have to drink my pill if I feel better	3	5
I do not have transport money to go to clinic	2	3
I am not responsible for taking my medication	1	2
I am too old to go to the clinic by myself	1	2
The medicine makes me feel worse	1	2
I don’t have to take my medication if am going to the hospital	1	2

**Total**	**58**	**100**

*Source*: Author’s own work

Table 4 shows the reasons for poor adherence to healthy lifestyle recommendations. Most of the participants, 20 (29%), stated that the main reason for not adhering is that they were too old to do exercise, 15 (22%) had no specific reason, 9 (13%) struggled to motivate themselves and 7 (10%) simply said they forgot to follow the advice.

**TABLE 4:** Reasons for poor adherence to healthy lifestyle recommendations.

Reasons	Number of respondents	% of respondents
I am too old	20	29
There is no specific reason for me not to	15	22
I struggle to motivate myself	9	13
I forgot	7	10
The lifestyle changes make me feel worse	5	7
I do not have enough time for that	4	6
Work did not allow me to carry out the changes	3	4
I am not responsible for carrying out the changes	2	3
I do not have to adhere to lifestyle changes if I feel better	2	3
I do not believe that it will help me	1	1
Has an amputated foot	1	1

**Total**	**69**	**100**

*Source:* Author’s own work

Adherent patients stated that the following factors aided them in adhering to treatment: timing the taking of medication with meals (68%), setting cell phone reminders (14%) and using the assistance of a treatment supporter (8%), while the remaining respondents reported using other means.

## Discussion

In this study, the level of self-reported adherence to diabetes medication was 70%, which is higher than that reported in most studies. For instance, a systematic review by Cramer^12^ found a very wide range of adherence levels (between 36% and 93%), and adherence levels depended largely on the method of assessment used. This result was buttressed by another study, which estimated poor adherence to be between 30% and 50% irrespective of disease, prognosis or setting.^13^ Furthermore, Manan et al., in their study of adherence among patients with type 2 diabetes, found a self-reported adherence level of 48%.^14^ Similar studies tended towards the high end of the spectrum: Kalyango et al.^6^ and Gelaw et al.^15^ placed adherence at 71.1% and 72.2%, respectively. The high adherence in our study was most likely because of the fact that it was self-reported; the sampling was biased as only patients who came to collect treatment – thus adhering – participated in the study and, furthermore, a tendency to give pleasing answers could also have influenced the results.

Patient adherence to a prescribed regimen of oral hypoglycaemic agents is generally low and difficult to maintain, even in populations with adequate access to healthcare and drug coverage.^4^ Therefore, it is most likely that, in our case, the assessment method used overestimated the true value of adherence in our setting. The subjective nature of the method of assessment, where the response was dependent solely on the power of recall, attitude and trustworthiness of the patient, might have contributed to this high value. This notion is reinforced by Prado-Aguilar et al.^16^ who stated that patient self-reporting tends to overestimate adherence.

According to Schectman et al.,^17^ adherence to appointments, independent of frequency of visit, was a strong predictor of diabetes metabolic control. Similarly, in our study, of the respondents who kept their appointments, 98% reported adherence to treatment. From our data, other significant predictors of good adherence were an age below 50 years (*p* = 0.028) and being employed (*p* = 0.018). This tendency is confirmed by the literature, where younger, employed and educated patients were found to adhere better to treatment, although not always proven to be statistically significant.^12,13^ It is important to note that the majority of our patients were older than 50 years and unemployed, indicating that it is important to design appropriate health services to assist these patients in improving their adherence to treatment.

Our study found that strategies to remember medication included taking it at meal times, setting a reminder on a cell phone and by soliciting the help of a treatment supporter. On the contrary, Littenburg et al.^18^ showed that the most popular aid to treatment adherence was the day of the week pill box, keeping medicines in a special place and associating medicine with a daily event such as a TV show or a meal (the last mentioned being similar to our findings). However, differences in our respective study populations could account for this. Levels of education and socio-economic and cultural circumstances were amongst the main difference between respondents in our study and those of the study by Littenburg et al. Furthermore, the findings regarding taking medication with a meal are confirmed by Schectman et al., who also found that taking medication during meal times had an advantage.^15^

With regards to lifestyle factors, a study measuring adherence and barriers to lifestyle recommendations among patients with high cardiovascular risk factors in Kuwait found that 64.4% of participants were not participating in regular physical exercise. The main barriers to physical exercise programmes were a lack of time (39%), co-existing diseases (35.6%) and adverse weather conditions (27.8%).^19^ In our study, old age was the most common reason stated for not adhering to recommended lifestyle changes, and therefore, it is recommended that the exercise advice should be appropriate for patients’ age and environment, an example of a suitable physical exercise activity being walking. It is worthy to note that in the Limpopo Province the aged patients and patients with chronic illnesses formed soccer teams to encourage exercise, practising two or three times per week and competing amongst each other. This strategy is keeping them motivated while enjoying the exercise.

## Limitations of the study

The assessment of adherence was dependent on the memory and subjectivity of the participants, as our study relied on self-reported data. As self-reported adherence is usually higher than actual adherence, we suspect that actual adherence to diabetes management in these patients may be even lower than the reported level. Patients attending the hospital for medication constituted a biased study population as they were already complying with expectations. Further bias was possibly introduced during the interviews, as patients could have been culturally more likely to report what they perceived the interviewer preferred.

## Conclusions and recommendations

Although our study revealed a possibly biased, higher than expected adherence level, it is clear that most of our older and unemployed patients are prone to struggle with adherence to the treatment and lifestyle adjustments required for diabetes. Patients who miss their appointments also need additional support to adhere to treatment.

To address this, we recommend that the quality of services at primary care facilities should be improved by making access easier for older and unemployed patients living far from the hospital, by avoiding drug shortages and by involving a multidisciplinary team in the management of patients with diabetes and efforts should be made to improve patient education and to form appropriate support and exercise groups to encourage patients in their lifestyle adjustments.

Further research is needed to assess whether self-reported adherence corresponds to actual adherence and to the metabolic control of the patients. Operational research to develop cost-effective ways to ensure optimal and quality care for patients living with diabetes and other chronic illnesses will contribute significantly to improved health outcomes.
